# Social connectedness is associated with food security among peri-urban Peruvian Amazonian communities

**DOI:** 10.1016/j.ssmph.2018.02.004

**Published:** 2018-02-23

**Authors:** Gwenyth O. Lee, Pamela J. Surkan, Jon Zelner, Maribel Paredes Olórtegui, Pablo Peñataro Yori, Ramya Ambikapathi, Laura E. Caulfield, Robert H. Gilman, Margaret N. Kosek

**Affiliations:** a Department of Epidemiology, School of Public Health, University of Michigan, M5071 SPH II, 1415 Washington Heights, Ann Arbor, MI 48109-2029, USA; b Department of International Health, the Johns Hopkins Bloomberg School of Public Health, Baltimore, MD, USA; c Asociación Benéfica PRISMA, Iquitos, Perú; d Department of Global and Population, Harvard T.H. Chan School of Public Health, Boston, MA, USA

**Keywords:** Peru, Food security, Social capital, Social networks

## Abstract

**Background:**

Food insecurity is a major global public health issue. Social capital has been identified as central to maintaining food security across a wide range of low- and middle-income country contexts, but few studies have examined this relationship through sociocentric network analysis.

**Objective:**

We investigated relationships between household- and community-level social connectedness, household food security, and household income; and tested the hypothesis that social connectedness modified the relationship between income and food security.

**Methods:**

A cross-sectional census with an embedded questionnaire to capture social relationships was conducted among eleven peri-urban communities. Community connectedness was related to study outcomes of food security and per-capita income through regression models.

**Results:**

Of 1520 households identified, 1383 were interviewed (91.0%) and 1272 (83.9%) provided complete data. Households in the youngest communities had the most total contacts, and the highest proportion of contacts outside of the community. Household income was also associated with more outside-community contacts (0.05 more contacts per standard deviation increase in income, p<0.001).

Less food secure households reported more contacts nearby (0.24 increase in household food insecurity access scale (HFIAS) for each additional contact, p<0.001). After adjusting for household-level socioeconomic status, membership in an older, larger, and better-connected community, with a greater proportion of residents engaged in rural livelihood strategies, was associated with greater food security (-0.92 decrease in HFIAS for each one-unit increase in community mean degree, p=0.008). There was no evidence that social connectedness modified the relationship between income and food security such that lower-income households benefited more from community membership than higher-income households.

**Conclusions:**

Although households reported networks that spanned rural villages and urban centers, contacts within the community, with whom food was regularly shared, were most important to maintaining food security. Interventions that build within-community connectedness in peri-urban settings may increase food security.

## Highlights

• Community characteristics explained 5.5% of the variability in household food security.
• Better-connected, older, and larger communities were positively associated with food security.
• Community connectedness did not modify the relationship between household income and food security.

## Introduction

The role of social connectedness in protecting food security is of multi-disciplinary interest, spanning the fields of nutrition, public health, anthropology, sociology, and international development. Food sharing is a deeply ingrained social activity, and food-sharing networks have been studied for their role in building and maintaining cultural identity and social bonds (Koster and Leckie, 2014, Nolin, 2010, Trostle et al., 2007). These reciprocal relations and interactions may also increase household and community-level resilience by helping to maintain food security during periods of seasonal scarcity or following climatic or economic shocks (Adger, 2003, Hadley et al., 2007, Sherman et al., 2016). Conversely, chronic pressure on food resources that reduces food-sharing may erode social capital (Hadley et al., 2012, Sherman et al., 2016).

Many studies have reported that social support and social capital are central to promoting household-level food security in low and middle-income countries (Diaz et al., 2002, Martin et al., 2004). However, in these reports, social support and capital are often characterized through egocentric or proxy measures of support (for example, by asking a respondent to describe contacts who might provide them with support or membership in social organizations). These approaches may therefore overlook community-level effects, or produce counter-intuitive findings as needier households reach out to those individuals or groups that can help to address those needs (Isquith-Dicker, 2016). While several studies have used network analysis to examine the extent to which broader community structures function to mobilize resources towards vulnerable households, most have characterized only a single community (Koster and Leckie, 2014, Mertens et al., 2015, Nolin, 2012). However there is also evidence that some communities are more efficient than others at promoting the food security of their members (Diaz et al., 2002, Hadley et al., 2007). Social connectedness may also interact with household wealth to offer protection against food insecurity (Hadley et al., 2007), and better-connected communities may be more efficient at allocating resources towards the most vulnerable households (Diaz et al., 2002).

In the Peruvian Amazon, informal food sharing between households has been demonstrated to represent a key food security coping strategy. Other community-based practices intended to help households cope with economic scarcity such as *parilladas* (a chicken barbeque where plates are sold to neighbors, family and friends to raise money, are also common (Ambikapathi et al., 2018). In this context specifically, as in other sites throughout tropical Latin America, food security, and measures of social capital and social support, have been linked to child nutritional status (Fernández-Concha, Gilman & Gilman, 1991) as well as other indicators of child health (Surkan et al., 2012, Surkan et al., 2007), reinforcing the importance of these mechanisms to population health in this context. Qualitative work in the region has also highlighted the role of food sharing as a strategy to maintain household food security following environmental shocks such as flooding. However, this work also suggests that the strength of these networks is in decline as a result of insufficient resources and cultural change (Sherman et al., 2016).

To test the hypothesis that social networks are associated with food security, we characterized the social networks of eleven Peruvian Amazonian communities, considering risk factors at both the household and the community level. Specifically, we tested the hypotheses that (1) community-level social connectedness is positively associated with household food security; (2) community-level social connectedness is positively associated with monetary household income; and (3) that the relationship between community-level connectedness and food security is modified by household income (specifically, that lower-income families benefit more from social connectedness than higher-income families).

## Materials and methods

**Fig. 1:**
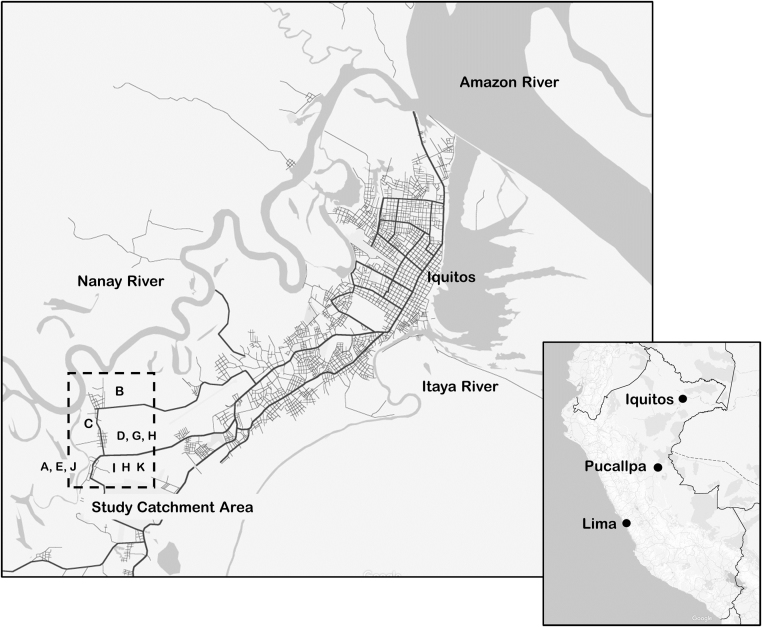
Location of study communities relative to the city of Iquitos, Peru: The study communities are located approximately 15 km from the city of Iquitos and were, at the time of the data collection, connected to the city by two dirt roads.

A detailed description of the study site is provided elsewhere (Yori et al., 2014). In brief, the study catchment area consisted of a cluster of peri-urban communities located approximately 15 km from the city of Iquitos, Peru, close to the *Nanay* river. The boundaries of the catchment area were defined by access: at the time of data collection, all communities were accessible to each other by foot, while traveling to the outskirts of Iquitos (or to other nearby peri-urban communities) required about 45 minutes travel via car or motorcycle on an unpaved road (Fig. 1). The communities were of varying ages (from over 50 years old to less than a year old). Residents of these communities report heterogeneous participation in “rural” livelihood strategies dependent on natural resources, and “urban” livelihood strategies. Common “rural” activities included farming, fishing, participation in the lumber industry and extended up-river trips to gather natural materials (such as ‘*palos redondos*’ hardwood used in construction, ‘*irapay’* a type of thatch used in making roofs, and *‘aguaje’* a popular local fruit), that were then brought to the city to sell. Common “urban” activities included moto-taxi driving, brick-making, and small-business ownership (Yori et al., 2014).

Our study design involved a census that every household in the study catchment area was invited to participate in. The study was conducted from May to August 2015. The boundaries of each community were defined based on the consensus of study fieldworkers and residents. Because all but the oldest of the communities had been formed through the organized occupation of previously vacant public or privately-owned land, and because community members work together to obtain legal title to their properties (Lee et al., 2016), there was little disagreement about where community boundaries lay. Although certain communities were additions to (newer neighborhoods of) older communities, they were considered distinct because they had been settled at a later time and by a different wave of inhabitants.

Households were defined as groups of individuals eating out of the same pot, and were enrolled with the written informed consent of the male or female head of the household. In each participating household, the full names of each household member as well as their ages and genders were recorded. Subsequently, targeted individuals within the household (both the male and/or female head) were separately invited and consented to participate in detailed individual interviews to collect further information on the variables described below. Household food security was collected only from the female head (defined as the person primarily responsible for preparing food for the household), while household socioeconomic, demographic, and livelihood indicators were preferentially collected from the head of the household (whether male or female), and social network information was collected separately from both the male and female head.

### Socioeconomic and demographic indicators

A socio-economic questionnaire previously developed by the study team (Kosek et al., 2008) was used to collect information about education, employment, land ownership, and sources of monetary income (through employment, remittances, and social programs), as well as household construction materials and access to improved water and sanitation. Because income in rural communities may also include non-monetary income such as income through directly consumed fish or agricultural products, the questionnaire was adapted to allow for the determination of a non-income based wealth index, the Progress Out of Poverty (PPI) index (the development of the Peruvian PPI and its relation to other country-specific PPIs is reported by Schreiner and colleagues (Schreiner, 2012), used to estimate the likelihood that a household has expenditure below the poverty line (Desiere and W D’Haese, 2015, Schreiner, 2012). The PPI is widely used in low and middle-income countries for program evaluation purposes (Banerjee et al., 2015). Reported per-capita income was calculated based on the total income reported by all members of the household 14 years of age or older through all sources, divided by the number of individuals in the household (including children), and transformed to a Z-score relative to the overall distribution ((log household per capita income - mean log per-capita income) / standard deviation log per-capita income). Non-monetary sources of income (for example, direct consumption of fish or food from farms) were not included in this calculation.

### Food security

The food security questionnaire included the Household Food Insecurity Access Scale (HFIAS), a measure of food security developed by the Food and Nutrition Technical Assistance (FANTA) project for cross-cultural use (Coates, Swindale & Bilinsky, 2007). The HFIAS has previously been validated at the study site (Psaki et al., 2012). The HFIAS was summed into a single continuous variable according to standard procedures (Coates et al., 2007). Secondly, we measured household dietary diversity by asking about the prior day’s consumption of items within twelve food groups (grains, root vegetables, vegetables, fruits, meat, eggs, fish, legumes, dairy, fats, sugars, and other foods) (Swindale & Bilinsky, 2006), and summing across all potential food categories. Finally, we asked about the number of times the household had received food as a gift in the past (Ambikapathi et al., 2018).

### Livelihood Strategies

Land- and natural-resourced based (‘rural’) livelihood strategies were identified based on the reported occupation of any household member (any individual that defined their occupation as fishing, working in the lumber industry, or agriculture as well as whether the household owned a ‘*chacra’* (small farm) or a boat or canoe (generally used for fishing or farming up-river, these asset measures are not included in the PPI). A household reporting any of these was defined as engaging in rural livelihood activities. Any household that reported any adult working in public transportation (moto-taxi or bus), brickmaking, working for the local gas factory, owning a business, or working as a domestic employee or as a qualified professional, was defined as engaged in ‘urban’ livelihood activities.

### Social networks

Social network information was collected via a questionnaire developed for the study. This questionnaire included an initial name-generating step that consisted of asking each respondent eight questions intended to elicit social contacts who provided instrumental aid, emotional aid, and companionship (Marin & Hampton, 2006), and one question intended to elicit contacts with who might assist if the interviewee was concerned about not having enough food. The instrument intentionally did not instruct interviewees to limit themselves to individuals living in the same community or nearby. Based on the list generated, additional information was requested about each contact, including his/her full name, approximate age, gender, and residential location. Finally, interviewees were asked about the degree to which the contact was a source of food support (‘*If you were concerned about having enough food, could you look for help from this person?*’), with possible responses ranging on a rating scale of “never” (0) to “always” (5). and the degree to which the contact was a source of stress (‘*How often does this person ask too much of you?”)*, with answers on the same scale.

### Formation of community networks

A probabilistic record linkage (PRL, also known as fuzzy-matching) algorithm was developed to link contacts (interviewee-reported full name, sex, and approximate age of contact) reported to be living in the study catchment area to their census record (name, age, and sex as reported for all household participants through the census) (Méray, Reitsma, Ravelli & Bonsel, 2007). This method has previously been used in risk network studies in the United States, where most interviewees were found to report contact characteristics accurately (Young et al., 2016). Because our interest was in inter-household, rather than intra-household support, only relationships between individuals living in different households were considered. Network data were aggregated to the household level by combining the contact list of the head of the household with the contact list of the food-preparer of the household. Because this approach may have caused households where the head of the household and the food-preparer of the household were the same person to be under-sampled (typically female-headed households, which represented 19.0% of the final sample), sensitivity analyses were conducted in which the household-level network was also constructed based only on the network of the head of household or only on the network of the food preparer. Further details of the PRL procedures are reported in Appendix 1.

### Household level network characteristics

The total number of reported number of contacts was calculated for each household. This was subdivided into the proportion of contacts reportedly 1) living in the study area that were not successfully matched to a census record via the PRL algorithm, 2) the proportion reportedly living in Iquitos, in another urban center (most frequently Lima, followed by the city of Pucallpa), and 3) in a rural area (almost exclusively small communities in Loreto) (see Fig. 1). For each household, the food security score of the most food-secure contact (lowest reported HFIAS score among all contacts identified), the education of the most educated contact (greatest years of education completed, among all contacts identified), and the degree of stress caused by the most stressful contact were also calculated.

### Community level network characteristics

Gephi 0.9.1 software was used to visually examine the network of each community and to calculate the community mean degree. Other community-level variables considered included community size (total number of households), community age, mean kin degree (the mean number of close or extended family members nominated living in the same community as the respondent), mean per-capita income, and the proportion of community households participating in rural livelihood activities.

## Statistical methods

*To examine the relationship between community connectedness and food security,* tobit regression models were constructed where the dependent variables were (i) food security, as measured by the continuous HFIAS scale and (ii) dietary diversity. Tobit models yield unbiased estimates when the dependent variable is truncated (Hsiao, 2003), as is the case here (Desiere et al., 2015) (HFIAS is truncated below 0 and above 27; dietary diversity is truncated below 0 and above 12). To adjust for potential household-level confounders with a known relationship to food insecurity (i.e. household socio-economic status), we also considered several household-level covariates with previously established relationships to food security. These included variables related to SES (per-capita income, PPI, the education of the head of the household, whether the household was female- or male-headed, and whether the household was engaged in rural livelihood activities, or urban livelihood activities, or a combination of both. Secondly, to distinguish characteristics of the local household connectedness from community connectedness, the out-degree of each household within or outside in the study catchment area, the food security status of the best available contact, the education level of the best available contact and the stress level of the most stressful reported contact) were considered. Finally, community-level connectedness was included. We also examined the relationship between community connectedness and other community characteristics including age, size, mean per-capita income, mean PPI, and the proportion of community households engaged in rural livelihoods.

For all variables, a bivariate model including a community-level random intercept was constructed. Secondly, a full multivariable model based on inclusion of all theoretically justifiable variables was included, and then a final reduced multivariable model was constructed with non-significant household-level descriptors of SES or alter characteristics excluded. Community-level factors other than connectedness were considered individually in bivariate models (Supplemental Table 3), but, because these factors were strongly correlated with community connectedness, only the community connectedness was included in multivariable models. Dominance analysis(Budescu, 1993) was conducted to determine the relative importance of household- and community-level variables in the overall model fit.

*To examine the relationship between community connectedness and household income,* we used a similar approach to test the relationship between the same factors described above, and normalized household per-capita income. In the multivariable model, we did not adjust for factors related to household SES (head of household education or head of household age) because the purpose of this analysis was to understand the relationship between contact- and community-level characteristics and per-capita income.

*To test the hypothesis that community connectedness modified the relationship between per-capita income and food security*, we tested for interactions between per-capita income and community mean degree in both the HFIAS scale and dietary diversity models described above.

## Results

**Fig. 2:**
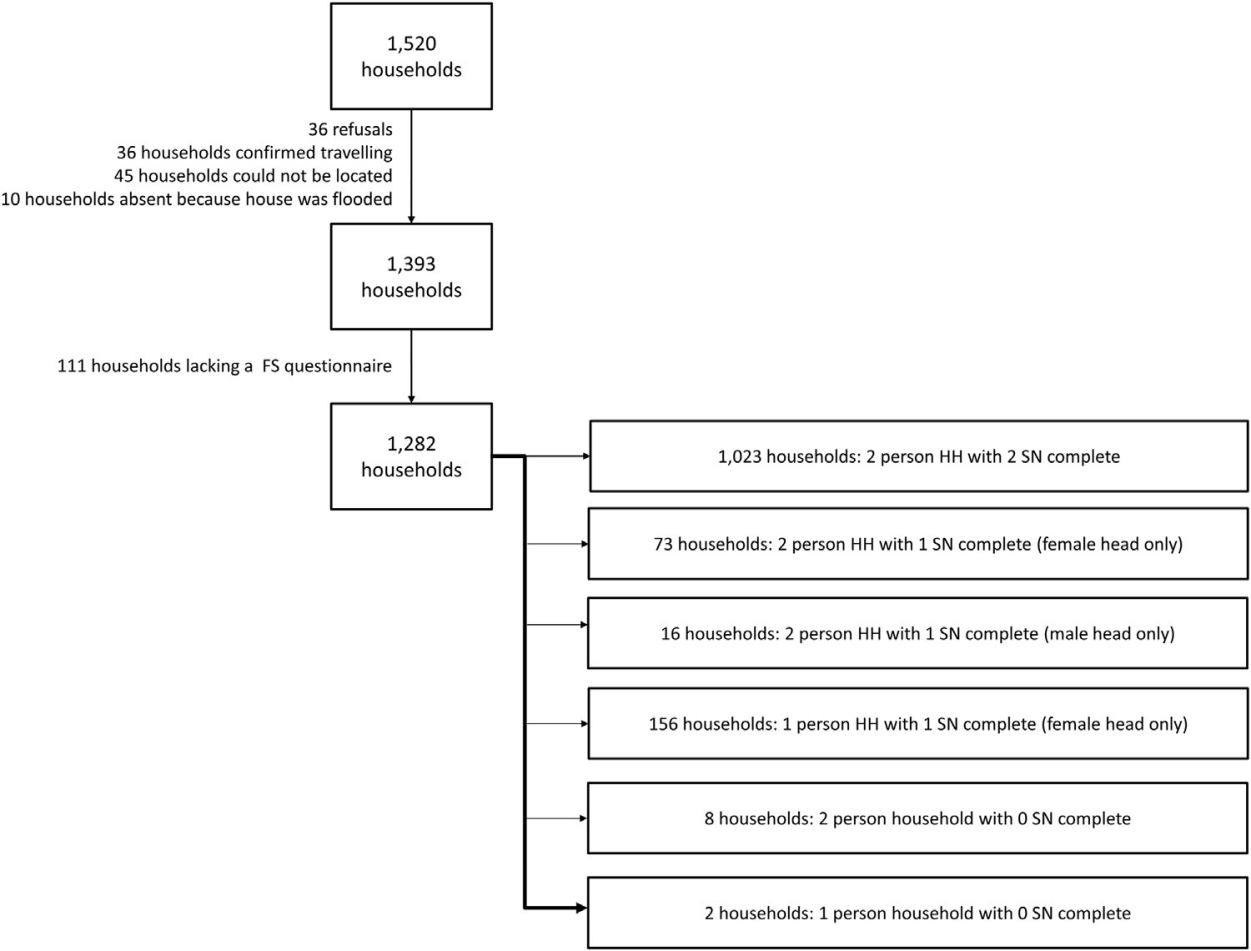
Flow chart describing study enrollment and data completeness: Of 1520 households identified within the study catchment area, 1393 were successfully enrolled in the study. Complete food security data were obtained in 1282 households, and complete food security and network data were obtained in 1272 households.

Of the eleven communities in the study catchment area, two were characterized as well-established (A and B), two were approximately a decade old (C-D), four were between two and five years old (E-H) and three had been formed in the past two years (I-K) (Table 1). 1520 households were identified, from which 1,393 were successfully interviewed (91.6%, Fig. 2). Of these, 1,282 (92.5%) households had a completed food security questionnaire available. Of the 1,282 households available, 1,023 (79.4%) had two completed network surveys (one from the male head and one from the female head), 245 (19.0%) had one completed network survey (156 of these were female-headed households with no male head), and 14 households had no network survey questionnaires completed (Fig. 2). Female heads of household reported fewer years of education than male heads of household (7.8 versus 8.8, p<0.001), and were less likely to be involved in rural livelihood activities (10.2% versus 35.6%, p<0.001), while two-person households with only one completed network survey were less likely to be involved in rural livelihood activities and relatively more food-secure than two-person households with two completed network surveys (Appendix 2).

**Table 1:** Characteristics of Reported Contacts, by Community Age: Shown here are characteristics of reported contacts in each of the eleven communities censused. Communities are divided into 4 sub-categories ranging from oldest communities (A and B) to youngest (K). (N=1393, except for statistics describing reported contacts which are based on N=1272).

		**>15-year-old communities**		**6 to 15-year-old communities**		**2 to 5-year-old Communities**				**1 to 2-year-old communities**		
	**TOTAL**	**A**	**B**	**C**	**D**	**E**	**F**	**G**	**H**	**I**	**J**	**K**
**Estimated Age of community**^a^	N/A	1959	1967	2003	2003	2010	2010	2011	2012	2013	2013	2014
**Total Individuals**	6035	669	2313	363	780	227	85	218	329	179	445	427
**Total Households**	1393	162	503	81	169	59	20	44	80	40	116	119
**Total Contacts nominated per HH Median (IQR)**	6 (4, 8)	7 (5, 9)	4 (3, 6)	5 (4, 7)	7 (5, 9)	7 (5, 9)	6.5 (5, 8)	6 (4.5, 8.5)	7 (5, 10)	9 (5, 12)	6 (4,5 9.5)	8 (5, 10)
**Percent of total contacts within (versus outside) catchment area**	68.9%	80.6%	78.3%	76.1%	72.7%	75.6%	67.3%	82.3%	42.5%	41.9%	48.9%	37.3%
**Head of household education Mean (SD)**	8.8 (4.1)	7.8 (4.1)	8.7 (4.1)	8.1 (3.9)	9.2 (4.3)	9.3 (3.9)	8.8 (3.8)	7.8 (4.3)	9.8 (3.6)	9.5 (3.7)	10.0 (3.8)	8.6 (4.0)
**Percent of male-headed households**	88.6%	87.0%	90.3%	93.8%	88.2%	86.4%	100.0%	90.9%	90.0%	90.0%	82.8%	83.2%
**Percent of households with rural livelihoods**	21.8%	25.3%	28.6%	19.8%	23.1%	15.3%	20.0%	43.2%	10.0%	15.0%	7.8%	6.7%
**Percent of households with urban livelihood**	42.2%	33.3%	53.1%	37.0%	34.3%	40.7%	40.0%	25.0%	50.0%	22.5%	37.1%	37.0%
**Per-capita income Peruvian Nuevo Soles/person/month Median (IQR)**	55.8 (36.2, 93.0)	62.0 (38.8, 96.9)	54.3 (31.0, 80.6)	51.7 (31.0 103.3)	52.7 (35.3, 93.0)	62 (31.0, 77.5)	53.7 (34.1, 84.0)	46.5 (24.8, 66.7)	62.0 (43.9, 93.0)	51.7 (30.8, 101.8)	62.0 (45.0, 98.7)	62.0 (41.3, 95.5)
**HFIAS score Mean (SD)**	11.0 (5.2)	12.3 (4.1)	8.5 (5.7)	10.8 (5.0)	12.2 (3.9)	12.3 (2.9)	12.6 (4.3)	12.3 (4.6)	12.3 (3.7)	13.9 (4.0)	12.3 (4.2)	13.8 (4.6)
**Dietary diversity Mean (SD)**	9.0 (2.0)	8.8 (1.7)	9.3 (2.7)	8.6 (1.9)	8.9 (1.6)	8.4 (1.3)	8.6 (1.3)	8.4 (1.5)	9.1 (1.6)	8.9 (2.1)	9.1 (1.8)	9.1 (1.6)

Shown here are characteristics of the households in each of the eleven communities censused. All statistics are based on a sample size of 1393, except for food security statistics, which are based on a sample size of 1282.

a Estimated as the 10th percentile of the reported arrival year

Further details of community membership as it relates to the history of community formation are described in Appendix 3.

### Characteristics of reported contacts

Across communities, 16.1% of households reported at least one rural contact, 60.9% reported at least one urban contact (in Iquitos or elsewhere), and 12.1% of households reported both rural and urban contacts. Households in the newest communities reported the highest number of total contacts, but also the highest proportion of contacts living outside their own community (both living in Iquitos and living in more rural communities). In contrast, households in the oldest communities reported the fewest total contacts, although the highest proportion (and the highest absolute number) of contacts living locally (in the same community or in the study catchment area) (Table 1).

58.1% of all contacts nominated were described as immediate or extended family, and 14.8% were immediate family. Households in the oldest communities reported the most immediate family contacts. The percentage of contacts identified as family also increased with distance: 37.9%, 45.7%, 61.6%, 85.0%, and 76.6% of within-community, study catchment area, Iquitos, other urban area, and other rural area contacts were identified as family.

**Fig. 3:**
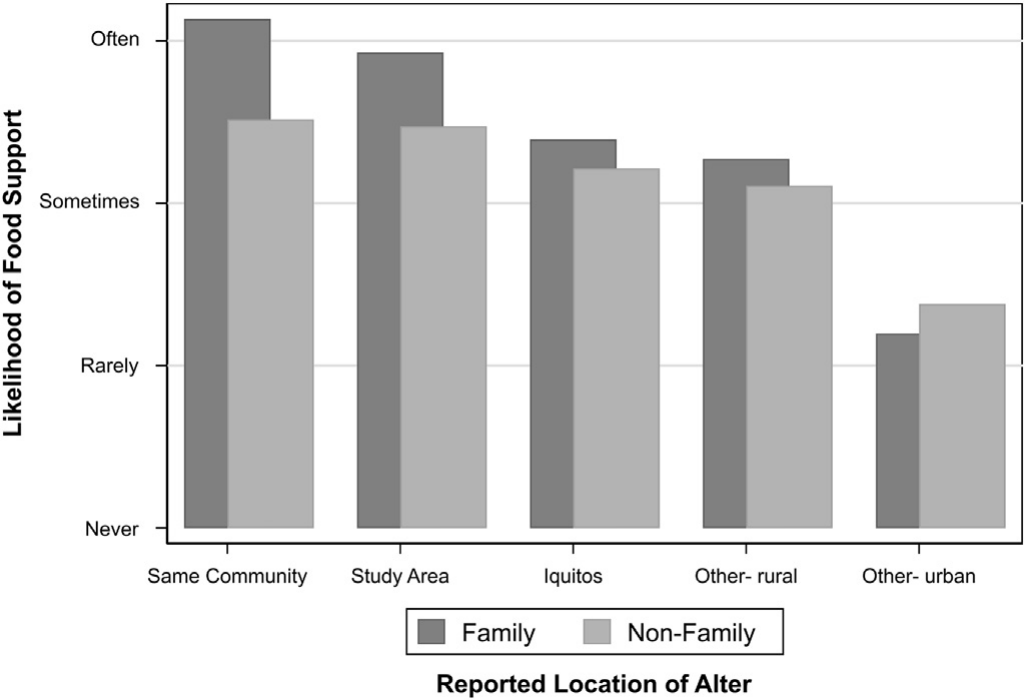
Relationship between the degree of food support reported provided by contacts, and the location and kinship of those contacts. The frequency of food sharing was determined based on the question “*If you were concerned about having enough food, could you look for help from this person?*”, with possible responses ranging on a rating scale of “never” (0) to “always” (5). Bar heights correspond to mean rating scale responses and the X-axis corresponds to the reported location of the contact. The “Other-rural” category of alter location was almost entirely comprised of small communities in other parts of Loreto; “Other-urban” was comprised of Lima (56.2%), Pucallpa, which is a large Amazonian city (8.4%), and other Amazonian cities (e.g. Tarapoto, Nauta, Requena).

More food support was reported from contacts living in the same community or nearby, and slightly more food support was reported from immediate or extended family contacts versus non-family contacts (Fig. 3).

### Community characteristics

**Fig. 4:**
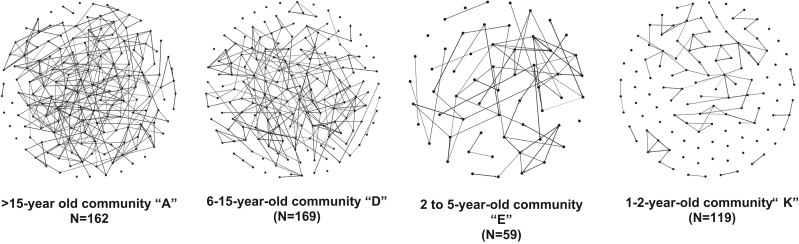
Community connectedness decreases with community age: Networks of four of the eleven study communities are shown below. Households are represented by points and lines between household represent contacts nominated between households. All eleven study communities are shown in Supplemental Figure 1.

Community age and community connectedness were strongly correlated (Fig. 4**)**. Correlations between community age, size, connectedness (mean degree), mean kin degree, and the proportion of households in the community engaged in a rural livelihood activity, ranged from 0.41–0.99 (Spearman’s ρ) (Fig. 4**)**.

Although positively correlated at the household level (Spearman ρ=0.45, p<0.001), mean community per-capita income and mean PPI were not significantly correlated (Spearman ρ=0.19, p=0.5739). The mean per-capita income was unassociated with community age (Spearman ρ=0.24, p=0.4692), while the mean PPI was positively associated with age (rho=0.6130, p=0.0449), due to a combination of more provisional and smaller houses and decreased asset possession in the newer communities (and despite slightly greater education and smaller household sizes). Because of these correlations, only household per-capita income was included in multivariable regression analyses.

### Associations with food security

Factors related to household socioeconomic status were associated with a lower HFIAS score (better food security) Each year of head of household education was associated with a 0.25 lower HFIAS (p<0.001); each ten years of age associated with a 0.31 greater HFIAS, and each standard deviation increase in household income was associated with a 1.38 lower HFIAS score (Supplemental Table 4). Engagement in an urban livelihood was not associated with the HFIAS score, but was positively associated with dietary diversity (Table 2**,** Supplemental Table 4). Engagement in a rural livelihood activity was associated with a higher HFIAS score in bivariate models, but after adjusting for household SES, this relationship was attenuated (Table 2**)**. There was also no evidence that increased livelihood diversity (engagement in both rural and urban livelihoods simultaneously) promoted food security or dietary diversity. More contacts living nearby was associated with greater HFIAS scores (reduced food security), while more contacts living further away was associated with greater dietary diversity.

**Table 2:** Household-, contact-, and community-Level factors associated with food security, dietary diversity, and per-capita income in multivariable regression models: Food security is represented by the continuous HFIAS score, dietary diversity by a 12-food group score, and per-capita income as a standardized Z-score relative to the overall logged distribution. The equivalent bivariable models are shown in Supplemental Table 3.

	**Difference in HFIAS (β coefficient (95% CI) p-value**	**Difference in Dietary Diversity (β coefficient (95% CI) p-value**	**Difference in Per-Capita Income (per Z-score) (β coefficient (95% CI) p-value**
***Household-level covariates***			
**Head of household education (per year education)**	-0.15 (^a^-0.23, -0.07) (p<0.001)	0.09 (0.05, 0.12) (p<0.001)	Na
**Female headed household (REF=male)**	Na	Na	Na
**Head of household age**^a^	0.03 (0.01, 0.06) (p=0.003)	0.01 (0.00, 0.02) (p=0.012)	Na
**Household income (per Z-score)**	-0.77 (-2.04, -0.50) (p=0.232)	0.34 (-0.24, 0.92) (p=0.250)	Na
**Presence of rural livelihood activity**	Na	Na	-0.28 (-0.44, -0.12) (p<0.001)
**Presence of urban livelihood activity**	Na	0.34 (0.08, 0.60) (p=0.011)	-0.03 (-0.17, 0.11) (p=0.671)
**Presence of both rural and urban livelihood interaction**	Na	Na	0.37 (0.11, 0.62) (p=0.005)
		
***Direct Contact-level characteristics***			
**Mean degree within catchment area**	0.24 (0.11, 0.37) (p<0.001)	0.05 (-0.02, 0.011) (p=0.139)	-0.02 (-0.05, 0.01) (p=0.097)
**Mean degree outside of catchment area**	0.05 (-0.06, 0.22) (p=0.257)	0.09 (0.03, 0.16) (p=0.004)	0.05 (0.02, 0.08) (p<0.001)
**Food security score of best available contact**^b^	0.05 (-0.02, 0.11) (p=0.175)	Na	Na
**Education of best contact**^c^	Na	0.04 (0.01, 0.08) (p=0.014)	0.02 (0.01, 0.04) (p=0.010)
**Most stressful contact**^d^	0.25 (0.05, 0.45) (p=0.015)	0.11 (0.01, 0.20) (p=0.023)	0.04 (0.00, 0.09) (p=0.041)
		
***Community-level characteristics***			
**Community connectedness**	-0.92 (-1.60, -0.24) (p=0.008)	0.13 (-0.07, 0.20) (p=0.023)	0.05 (-0.02, 0.11) (p=0.148)
			
***Interaction term***			
**Community connectedness x household income**	-0.11 (-0.38, 0.15) (p=0.408)	-0.04 (-0.16, 0.09) (p<0.001)	Na
		
***Random Effect Parameters***			
**SD (cons)**	1.03 (0.52, 1.55)	0.24 (0.07, 0.41)	0.00 (-0.00, 0.00)
**SD (resid)**	4.73 (4.53, 4.94)	2.17 (2.07, 2.27)	0.98 (0.94, 1.02)

a Age=per 10 y, centered at 45 y

b Lowest reported HFIAS score among all contacts identified

c Greatest years of education completed, among all contacts identified

d the degree of stress caused by the most stressful contact (on a scale of 1–5 where one is least and five is most stressful)

After adjusting for household-level characteristics, community connectedness was inversely associated with HFIAS scores (better food security). There was no relationship between the mean per-capita income of the community and food security (Supplemental Table 3). Household-level SES explained 7.0% of the variability in HFIAS, characteristics of household contacts explained an additional 7.9%, and community connectedness explained 3.4%. After adjusting for all these factors, community membership still explained an additional 4.5% of HFIAS scores.

Similarly, household SES, contact characteristics, and community connectedness explained 3.7%, 1.5% and 0.5% of variability in the dietary diversity score, respectively, and community membership explained an additional 0.8% of variability in dietary diversity after adjusting for these factors.

### Associations with per-capita income

Households that reported higher incomes tended to have more contacts outside of the community (each additional contact associated with 0.05 Z-score increase in income, p<0.001). Households that reported higher incomes were also marginally more likely to report that their contacts were a source of stress (Table 2).

Households reporting a rural livelihood had lower per-capita incomes than those without (-0.27 Z-score decrease in income, p=0.001), but households with diverse livelihoods (both rural and urban simultaneously) had higher incomes than households reporting only one or the other, or neither. Community connectedness was not significantly associated with household income, and the community-level random intercept explained <0.01% of the variability in reported per-capita income.

### Interactions between community connectedness and household income

Interaction terms between household per-capita income and community connectedness were non-significant, suggesting that although community membership contributed to HFIAS scores and dietary diversity, it did not modify the relationship between per-capita income and these outcomes.

## Conclusion

While many studies have considered the impact of social support on health outcomes and food security in low and middle-income countries (Becquey et al., 2012, Kaschula, 2011), relatively few have employed sociocentric sampling designs (Perkins, Subramanian & Christakis, 2015) that allow connections amongst individuals or households to be mapped. Sociocentric studies of food-sharing networks have been reported in Indonesia, Ecuador, Nicaragua, Brazil and Arctic Canada (Collings, Marten, Pearce & Young, 2015; Koster & Leckie, 2014; Nolin, 2012, Nolin, 2010; Trostle et al., 2007), the final two of which examined impacts on food security (Collings et al., 2015, Mertens et al., 2015). Our study examines heterogeneity in the extent to which community networks act as a source of social capital to maintain food security (Diaz et al., 2002), comparing established to recently formed, peri-urban settlements. Given that as much as a third of the world’s population (UN-Habitat, 2012), and a quarter of the population in Latin American (Fernandes, 2011, Mac Donald, 2004), live in informal peri-urban communities, there are practical implications to understanding the behavior of social networks in this context. Our results suggest that households in recently-formed communities should be targeted for support, as they were found to be at disproportionate risk of food insecurity despite factors such as smaller household sizes, greater education, and per-capita incomes similar to those of households in older communities.

We did not find evidence that the relationship between community connectedness and food security was modified by household income (i.e. lower-income households did not benefit more from community connectedness than households with higher income); but this finding is supported by other studies (Hadley et al., 2007). Households with fewer resources also tend to have contacts with fewer resources, leading to a reduced capacity to mobilize support through their social networks when needed (Adams, 1993, Hadley et al., 2007, Lemke et al., 2003). A study of food-sharing in Indonesia also found that wealthy households tended to give less food, and receive more, than poorer households (Nolin, 2012). We also found that higher-income families reported more geographically distant contacts even after adjusting for household livelihood activities associated with travel, and were more likely to report that their contacts were a source of stress. Overall, our results suggest that the relationships between connectedness and food security were distinct from, and could not be explained by, the relationships between connectedness and monetary income.

Extended rural-urban networks have been reported throughout Amazonia, and may promote economic stability by allowing families to benefit from urban incomes while reducing dependence on exclusively urban employment and residence (Padoch et al., 2008). This phenomenon is sometimes referred to as ‘straddling’ (Flynn, 2005, Potts and Mutambirwa, 1990). We also observed extended geographic networks, but found that within-community support was more strongly associated with food security than the presence of remote contacts. Relationships amongst neighbors are associated with frequent contact, mutual awareness of problems, and easy delivery of support (Wellman, 1992), and fellow community members may be more adept at noticing and responding to transient food insecurity than contacts living further away. Food insecure households reported more contacts living nearby, suggesting that the need for support may have promoted engagement. However, membership in a better-connected community was associated with greater food security. Similarly, engagement in rural livelihood strategies was positively associated with food security at a community- but not a household-level, perhaps suggesting that the benefit of these strategies was shared amongst community members rather than reverting exclusively to the household.

Our study also had several limitations. First, although the boundaries of our catchment area were locally meaningful and reflective of travel times as well as our study hypotheses, the large number of contacts reported between communities and in nearby Iquitos emphasizes the extent to which these boundaries were also porous. Furthermore, we were only able to match ~88% of nominated contacts reportedly living within the catchment area. This is likely a result of the 9.0% of households that could not be censused (2.6% refusals and 6.4% probable travel), as well as inaccurately or incompletely reported names or limitations in the PRL algorithm. An additional limitation is that, among the 111 households that lacked a food security questionnaire, 71 were households comprised of single men living alone. In these instances, interviewees often reporting eating with family living nearby or purchasing their meals from street vendors and therefore may not, based on the definition of “household” that included individuals sharing meals, have considered to be independent units. However, this was not tracked consistently. Finally, as is the case for all cross-sectional studies, we are unable to draw conclusions about potential causality between food security, income, and network characteristics, in our sample.

We also found that community connectedness, community age, the mean number of within-community contacts who were described as relatives, and community participation in rural livelihoods were strongly correlated within our sample, limiting our capacity to disentangle the role of these factors in promoting food security. Some of these factors are more intervenable than others. For instance. programs that build social connections within newly-formed communities and provide mechanisms for rural to-urban migrants to bring existing knowledge and capacities into the urban sphere, may be particularly useful.

Our results suggest that larger, older, and better-connected communities contribute more to the household food security of their members, and that participation in rural livelihood activities may have spill-over benefits, by promoting food security at the community level. In contrast, households in newly formed communities are at disproportionate risk of food insecurity, despite factors such as smaller household sizes, greater education, and per-capita incomes similar to those of households in older communities. These results support the utility of programs that promote food security by increasing food availability and access, support the strengthening of social networks as an amplifying mechanism by which these foods become distributed throughout the wider group.
