# Supplementary material for: Social connectedness is associated with food security among peri-urban Peruvian Amazonian communities
Source: SSM Popul Health. 2018 Feb 23;4:254–62. doi: 10.1016/j.ssmph.2018.02.004 (PMC5976826; doi:10.1016/j.ssmph.2018.02.004)
Supplement: Supplementary file 1 — Supplementary material [file mmc1.docx]

**Supplemental materials:**

**Appendix 1: Description of probabilistic record linking (PRL) algorithm procedure**

The PRL algorithm used to match interviewee-reported contacts to individuals in the census record assigned greater matching weight when the reported first name and paternal last name (as compared to the maternal last name) of the contact matched the census record.

Only matches with a strength-of-match score of 0.65 were retained, in the case of multiple potential matches, the strongest match between the named contact and the census record was retained. To check the impact of the linkage procedure on the network, a sensitivity analysis was also conducted in which alternative contact networks were constructed by discarding imperfect matches at a rate proportional to their strength-of-match score (for example, if a named contact had a strength-of-match of 0.80 to another individual in the census record, it was retained 80% of the time). The percent disagreement between these alternative networks and the network constructed using only best available match, according to the PRL algorithm, was then calculated.

By this method, we were able to match 88% of nominated contacts reportedly living within the catchment area. Comparing the final contact network to alternative contact networks, 79.4% of matched contacts did not change (5^th^, 95^th^ percentile = 79.1%, 79.8%); 17.5% (17.3%, 17.7%) of contacts became unmatchable (i.e. there was only one strong match for this contact in the census record, and it was discarded), and 3.0% (2.7, 3.3%) of contacts were matched to a different census record (i.e. the contact was strongly matched to multiple census records and, after discarding the best available match, they were linked to a different record).

**Appendix 2:**

**Supplemental Table 1: Comparison of households with complete versus incomplete network questionnaires.**

|  | **TOTAL** | **Two-person household missing one network questionnaire**  **(N=89)** | **Female-headed household**  **(156)** | **Two-person household with two network questionnaires**  **(N=1023)** | **p-value** |
| --- | --- | --- | --- | --- | --- |
| **Total Contacts nominated per HH**  **Median (IQR)** | 6  (4, 8) | 2.9 (1.2)* | 3.9 (1.6)* | 6.7 (2.9) | <0.0001 |
| **Percent of total contacts within (versus outside) catchment area** | 68.9% | 72.9% | 68.2% | 68.6% | 0.4549 |
|  |  |  |  |  |  |
| **Head of household education**  **Mean (SD)** | 8.8  (4.1) | 9.6 (4.2) | 7.8 (4.6)* | 8.8 (2.9) | <0.0001 |
|  |  |  |  |  |  |
| **Percent of households with rural livelihoods** | 21.8% | 23.6%* | 10.2%* | 35.6% | <0.0001 |
| **Percent of households with urban livelihood** | 42.2% | 50.6% | 46.2% | 42.4% | 0.2553 |
| **Per-capita income**  **Peruvian Nuevo Soles/person/month**  **Median (IQR)** | 55.8  (36.2, 93.0) | 51.7  (37.2, 77.5) | 54.1  (31.0, 102.8) | 54.3  (34.9, 80.6) | 0.7242 |
| **HFIAS score**  **Mean (SD)** | 11.0  (5.2) | 9.6 (5.8)* | 11.4 (5.9) | 11.0 (5.0) | 0.0284 |
| **Dietary diversity**  **Mean (SD)** | 9.0 (2.0) | 9.0 (2.1) | 8.9 (2.1) | 9.0 (1.9) | 0.8099 |

*indicates that the variable was different in t-test (column 1 versus column 3 and column 2 versus column 3)

**Appendix 3: Community membership and history of community formation:**

Within the eleven communities in the study catchment area, two were characterized as well-established (A and B, both in existence for over a decade); three were approximately a decade old (C and D, where C was a newer addition to D), four were between two and five years old (G, F, H, I, where G and H were newer additions to D, E was a newer addition to A, and F was a newly formed, independent community), and three had been formed in the past two years (I, J, K*,* where J is a newer addition to A and I and K which are both independent)

Overall, the history of the community formation was reflected in the reported migration history of its members: for example, E was an addition to B and nearly 50% of its residents reported being born in B, and D was formed in 2002 by members of both A and B, and approximately 15% of heads of household reported being originally born in each of these communities. In contrast, few individuals from the most recently formed communities of H and K were originally from the study catchment area. Instead, these were comprised in approximately equal parts of individuals born in Iquitos (urban) and individuals born in rural, riverine communities (**Supplemental Table 1**).

**Supplemental Table 2: Migration History of Heads of Household, by community**

|  |  | **>15-year-old communities** | | **6 to 15-year-old communities** | | **2 to 5-year-old**  **Communities** | | | | **1 to 2-year-old**  **communities** | | |
| --- | --- | --- | --- | --- | --- | --- | --- | --- | --- | --- | --- | --- |
|  | **TOTAL** | **A** | **B** | **C** | **D** | **E** | **F** | **G** | **H** | **I** | **J** | **K** |
| **Head of household born in:** | | | | | | | | | | | | |
| **Community “A”** | 10.7% | 54.1% | 0.4% | 0.0% | 14.5% | 45.6% | 0.0% | 0.0% | 0.0% | 0.0% | 3.5% | 0.9% |
| **Community “B”** | 25.0% | 1.3% | 55.9% | 24.4% | 14.5% | 0.0% | 5.3% | 11.9% | 2.7% | 7.7% | 3.5% | 3.4% |
| **Iquitos:** | 21.8% | 13.2% | 11.5% | 19.2% | 17.0% | 22.8% | 42.1% | 21.4% | 47.3% | 33.3% | 43.9% | 40.7% |
| **Other urban area** | 12.2% | 11.9% | 9.5% | 14.1% | 13.9% | 14.0% | 5.3% | 9.5% | 10.8% | 7.7% | 21.1% | 15.3% |
| **Other rural area** | 30.4% | 19.5% | 22.7% | 42.3% | 40.0% | 17.5% | 47.4% | 57.1% | 39.2% | 51.3% | 28.1% | 39.8% |

**Appendix 4: Bivariable Regression Models**

**Supplemental Table 3: Household-, contact-, and community-level factors associated with food security, dietary diversity, and per-capita income in bivariable regression models.**

Shown here are the bivariable model equivalents of the multivariable models reported in Table 3.

|  | **HFIAS** |  | **Dietary Diversity** |  | **Per-Capita Income** |
| --- | --- | --- | --- | --- | --- |
|  | **Bivariate** |  | **Bivariate** |  |  |
| ***Household-level covariates*** | |  |  |  |  |
| **Head of household education** | -0.25  (-0.33, -0.19)  (p<0.001) |  | 0.08 (0.05, 0.11)  (p<0.001) |  | 0.05 (0.04, 0.07)  (p<0.001) |
| **Female headed household (REF=male)** | 0.16  (-0.70, 1.01)  (p=0.721) |  | -0.04 (-0.42, 0.34)  (p=0.83) |  | -0.03 (-0.20, 0.14)  (p=0.717) |
| **Head of household age*** | 0.31  (0.12, 0.50)  (p=0.001) |  | 0.02 (-0.06, 0.11)  (p=0.60) |  | 0.06 (0.02, 0.10)  (p=0.002) |
| **Household income** | -1.38  (-1.64, -1.11)  (p<0.001) |  | 0.31  (0.19, 0.43)  (p<0.001) |  | Na |
| **Rural livelihood** | 0.65  (0.02, 1.27)  (p=0.042) |  | -0.03 (-0.31, 0.25)  (p=0.828) |  | -0.19 (-0.31, -0.07)  (p=0.002) |
| **Urban livelihood** | -0.38 (-0.96, 0.19)  (p=0.188) |  | 0.42 (0.16, 0.67)  (p<0.001) |  | 0.14 (0.02, 0.25)  (p=0.021) |
| ***Direct contact level characteristics*** | |  |  |  |  |
| **Mean degree within catchment area** | 0.19 (0.07, 0.30)  (p=0.002) |  | 0.04 (-0.02, 0.10)  (p=0.205) |  | -0.03 (-0.05, -0.01  (p=0.005) |
| **Mean out-degree outside of catchment area** | -0.04 (-0.16, 0.08)  (p=0.511) |  | 0.09 (0.04, 0.15)  (p=0.001) |  | 0.05 (0.03, 0.07)  (p<0.001) |
|  |  |  |  |  |  |
|  |  |  |  |  |  |
| **Most stressful contact** | 0.23 (0.02, 0.44)  (p=0.28) |  | 0.12 (0.03, 0.22)  (p=0.010) |  | 0.03 (-0.01, 0.07)  (p=0.108) |
|  |  |  |  |  |  |
| **Food security score of best contact** | -0.00  (-0.07, 0.06)  (p=0.958) |  | -0.03 (-0.06, 0.00)  (p=0.069) |  | 0.00 (-0.00, 0.02)  (p=0.493) |
| **Education of**  **best contact** | -0.03  (-0.11, 0.04)  (p=0.395) |  | 0.06 (0.03, 0.10)  (p<0.001) |  | 0.02 (0.00, 0.03)  (p=0.010) |
|  |  |  |  |  |  |
| ***Community-level network characteristics*** | | | |  |  |
| **Community age** | 0.05 (0.01, 0.09)  (p=0.018) |  | -0.01 (-0.02, 0.00)  (p=0.086) |  | -0.00 (-0.00, 0.00)  (p=0.691) |
| **Community size (per 100 households)** | -0.96 (-1.32, -0.60)  (p<0.001) |  | 0.19 (0.13, 0.26)  (p<0.001) |  | -0.01 (-0.05, 0.03)  (p=0.570) |
| **Mean degree** | -0.89 (-1.61, -0.17)  (p=0.015) |  | 0.10 (-0.09, 0.30)  (p=0.304) |  | -0.02 (-0.08, 0.05)  (p=0.596) |
| **Proportion of kin contacts** | -12.07 (-23.32, -0.82)  (p=0.036) |  | 0.56 (-2.79, 3.90)  (p=0.745) |  | -0.51 (-1.65, 0.62)  (p=0.375) |
|  |  |  |  |  |  |
| **Mean per-capita income** | 0.05 (-0.03, 0.12)  (p=0.203) |  | -0.01 (-0.03, 0.01)  (p=0.458) |  | 0.01 (0.00, 0.01)  (p=0.006) |
| **% of HHs with rural livelihoods** | -5.95 (-11.8, -0.09)  (p=0.047) |  | -0.10 (-1.82, 1.63)  (p=0.913) |  | -0.57 (-1.02, -0.13)  (p=0.012) |
|  |  |  |  |  |  |
| **Factor 1** | -0.61 (-0.97, -0.26)  (p=0.001) |  | 0.08 (-0.03, 0.180)  (p=0.163) |  | -0.02 (-0.05, 0.02)  (p=0.313) |

**Appendix 5: Network diagrams of study communities**

**Supplemental Figure 1: Network diagrams of all eleven communities**

Shown here are networks for all eleven study communities (A-K in order of community age). Households are represented by points and lines between household represent contacts nominated between households.

| 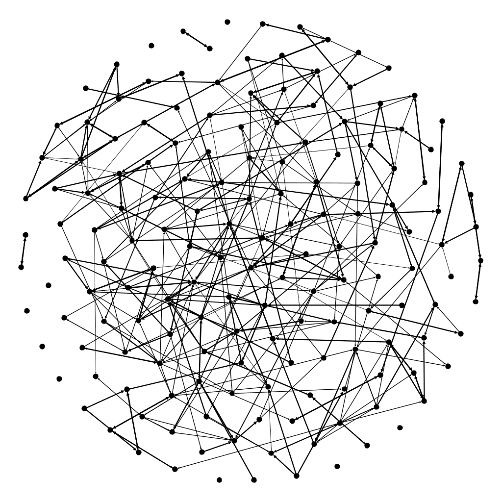  **>15-year old community “A” (N=162)** | 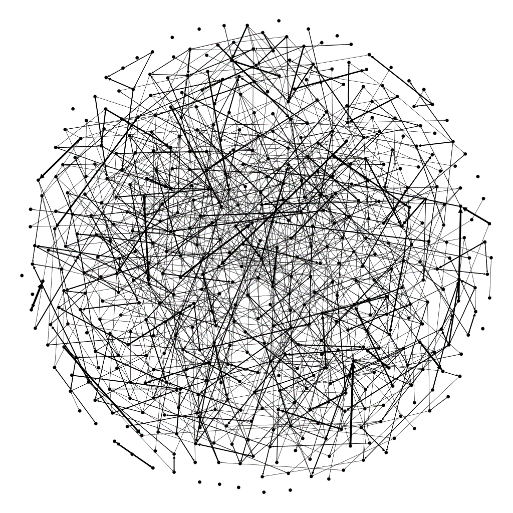  **>15-year old community “B” (N=503)** |
| --- | --- |
| 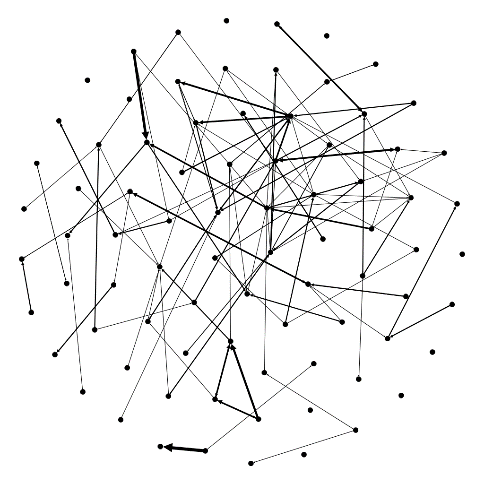  **6-15-year-old community “C” (N=81)** | 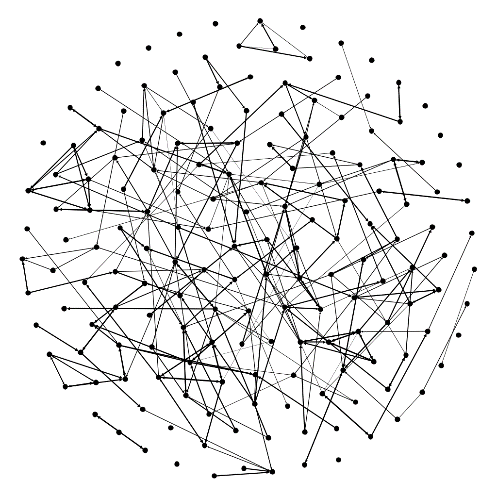  **6-15-year-old community “D” (N=169)** |
| 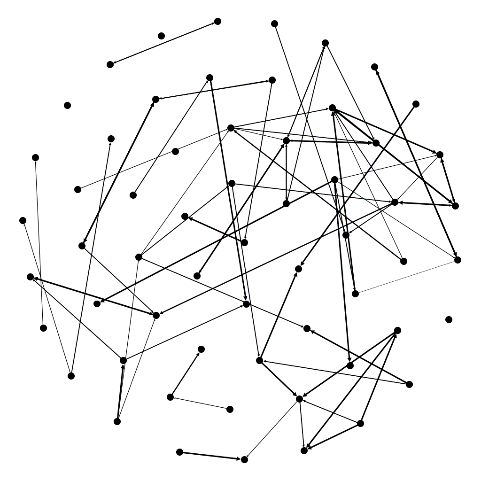  **2 to 5-year-old community “E” (N=59)** | 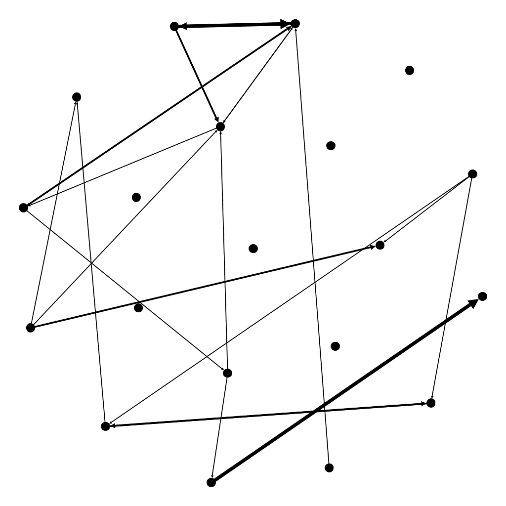  **2 to 5-year-old community “F” (N=20)** |
| 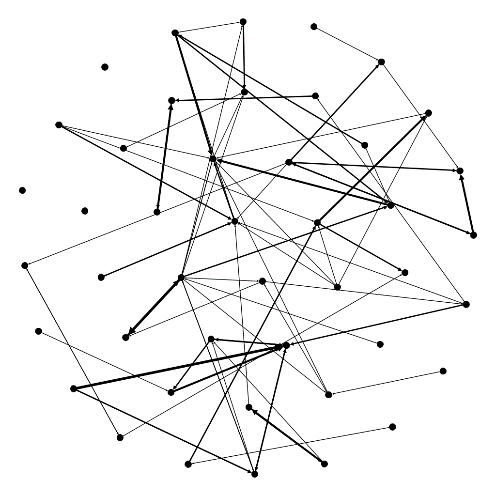  **2 to 5-year-old community “G” (N=44)** | 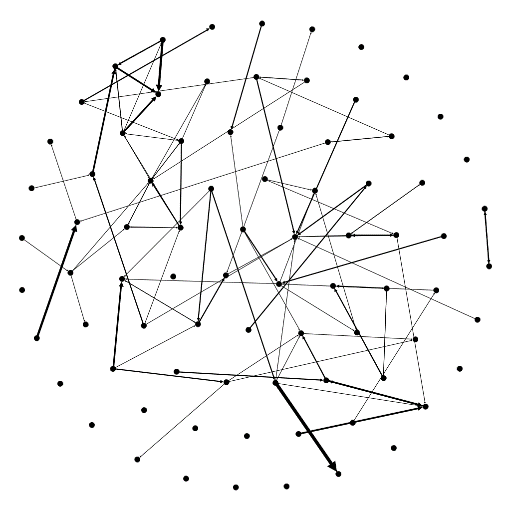  **2 to 5-year-old community “H” (N=80)** |
| 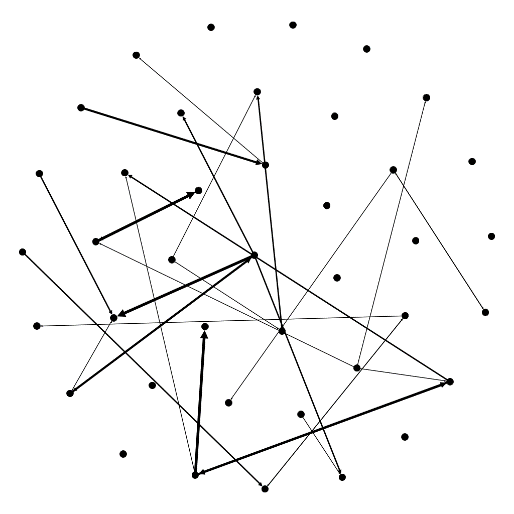  **1 to 2-year-old community “I” (N=40)** | 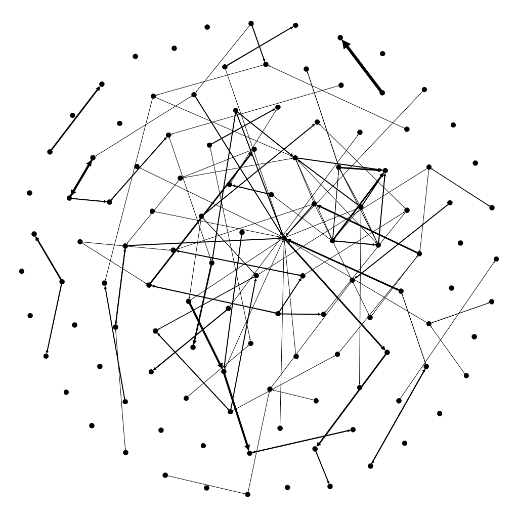  **1 to 2-year-old community “J” (N=116)** |
| 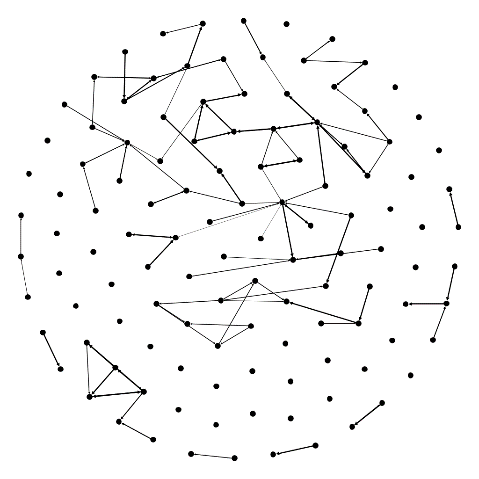  **1-2-year-old community “K” (N=119)** |  |
